# HEK293 Cell Line as a Platform to Produce Recombinant Proteins and Viral Vectors

**DOI:** 10.3389/fbioe.2021.796991

**Published:** 2021-12-13

**Authors:** Evan Tan, Cara Sze Hui Chin, Zhi Feng Sherman Lim, Say Kong Ng

**Affiliations:** Bioprocessing Technology Institute, Agency for Science, Technology and Research (A*STAR), Singapore, Singapore

**Keywords:** HEK293, human embryonic kidney cell 293, biotechnology, viral vectors, viral vectored vaccines, biotherapeutics and biologics

## Abstract

Animal cell-based expression platforms enable the production of complex biomolecules such as recombinant proteins and viral vectors. Although most biotherapeutics are produced in animal cell lines, production in human cell lines is expanding. One important advantage of using human cell lines is the increased potential that the resulting biotherapeutics would carry more “human-like” post-translational modifications. Among the human cell lines, HEK293 is widely utilized due to its high transfectivity, rapid growth rate, and ability to grow in a serum-free, suspension culture. In this review, we discuss the use of HEK293 cells and its subtypes in the production of biotherapeutics. We also compare their usage against other commonly used host cell lines in each category of biotherapeutics and summarise the factors influencing the choice of host cell lines used.

## Introduction

Production of complex biopharmaceutical products rely heavily on mammalian cell lines, with most therapeutic biopharmaceuticals produced in the Chinese hamster ovary (CHO) cells (Lalonde and Durocher, 2017). The use of mammalian production cells enables biologics with complex post-translational modifications (PTMs) to be produced. Complex biologics such as antibodies, growth and clotting factors require complex PTMs to ensure product stability and potency. However, not all biopharmaceutical products can be produced in CHO. Some recombinant proteins are made in HEK293 due to requirements in PTMs which cannot be met in CHO, and most therapeutic viral vectors are produced in HEK293. Since 2015, there have been seven HEK-derived products approved by the FDA (Table 1). Of these, six are cell and gene therapies (Mullard, 2016, Mullard, 2017; Mullard, 2018; Mullard, 2019; Mullard, 2020; Mullard, 2021) where the HEK293 cell line or its derivatives were used in the production of viral vectors. With the increasing number of cell and gene therapies being developed (Rittié et al., 2019; Lapteva et al., 2020), we will see a corresponding growth in the use of HEK293 in viral vector production.

**TABLE 1:** FDA-approved biologics produced in HEK293 since 2015.

Product Name	Properties	Approval	Reference
NUWIQ®	Recombinant anti-haemophilic factor	2015	U.S. Food and Drug Administration (2015); Mullard, (2016)
Voretigene neparvovec (Luxturna®)	AAV-based RPE65 gene therapy	2017	U.S. Food and Drug Administration (2017a); Mullard, (2018)
Axicabtagene cilolucel (Yescarta®)	CD19-directed CAR T therapy	2017	U.S. Food and Drug Administration (2017b); Mullard, (2018)
Tisagenlecleucel (Kymriah®)	CD19-directed CAR T therapy	2017	U.S. Food and Drug Administration (2017c); Mullard, (2018)
Onasemnogene abeparvovec (Zolgensma®)	AAV-based SMN gene theapy	2019	U.S. Food and Drug Administration (2019); Mullard, (2020)
Lisocabtagene maraleucel (Breyanzi®)	CD19-directed CAR T therapy	2021	U.S. Food and Drug Administration, (2021a)
Idecabtagene vicleucel (Abecma®)	BCMA-directed CAR T therapy	2021	U.S. Food and Drug Administration, (2021b)

## HEK293 Cells and Their Derivatives

The HEK293 cell line was established by transforming human embryonic kidney cells with sheared adenovirus type 5 DNA (Graham et al., 1977). Since then, many subtypes and derivatives have been established, with HEK293, HEK293-T, and HEK293-F frequently used in the production of biopharmaceuticals (Yuan et al., 2018). HEK293-T is a derivative of the HEK293 cell line, established by the expression of a temperature-sensitive SV40 T-antigen mutant (DuBridge et al., 1987). Expression of the T-antigen allows plasmids which carry the SV40 origin of replication to replicate when transfected into the cell (DuBridge et al., 1987). HEK293-F cells are GIBCO® brand cells, cloned from HEK293 and adapted to suspension culture in serum-free media.

Other notable HEK293 derivatives commonly used in recombinant protein production include HEK293-E and HEK293-6E. HEK293-E was established by the expression of Epstein-Barr nuclear antigen 1 (EBNA-1), which allows for episomal replication of plasmids with oriP. Similarly, the HEK293-6E cell line was established by the expression of a truncated EBNA-1, lacking Gly-Gly-Ala domain. HEK293-6E showed improved transient gene expression and cell growth compared to HEK293-E (Abaandou et al., 2021).

## Recombinant Protein Production in HEK293

Eukaryotic expression systems are used to produce complex recombinant protein with complex PTMs for proper protein function. The use of the HEK293 host cell eliminates issues of potential immunogenicity due to the presence of non-human PTMs (Durocher and Butler, 2009). Its ease of transfectability and relatively high protein productivity makes it a popular choice for small scale production of recombinant proteins for scientific studies (Abaandou et al., 2021) and its capability to adapt to a suspension free culture facilitates its use in large scale biotherapeutics production (Baldi et al., 2005; Dumont et al., 2015).

Because of its ease of transfection, HEK293 is widely used for transient gene expression (TGE). Recombinant proteins can be produced quickly by transfecting HEK293 with readily available reagents such as calcium phosphate or polyethylenimine (PEI). Such transfection processes have been demonstrated in stirred-tank bioreactors and have proved to be a good method for the rapid production of recombinant proteins for lab to pre-clinical use (Wurm and Bernard, 1999; Baldi et al., 2007). HEK293E and HEK293-6E is widely used in TGE as the use of plasmids with Epstein-Barr virus oriP significantly improves TGE (Pham et al., 2006). However, the main drawback of TGE in recombinant protein production is the need for large quantities of transfection-grade plasmid DNA.

Stable producer cell lines are preferred for the large-scale production of recombinant proteins. The most recent HEK-produced recombinant protein therapeutic, NUWIQ®, was produced in HEK293F cells. NUWIQ® is a recombinant coagulation factor VIII (FVIII). It is produced by transfecting HEK293F cells with a B-domain deleted human FVIII expression construct. After stable transfectants were selected, clones exhibiting optimal production were selected for use (Casademunt et al., 2012; Kannicht et al., 2015).

Protein production in producer cell lines can be increased by the use of gene amplification technology (Lai et al., 2013). Use of the glutamine synthetase (GS)-mediated gene amplification and selection system has been demonstrated in HEK293 cells (Yu et al., 2018; Chin et al., 2019), and could improve recombinant protein production (Chin et al., 2019). By coupling the expression of the recombinant human erythropoietin with glutamine synthetase in a bicistronic vector, erythropoietin expression was significantly improved after the sequential increase of methionine sulfoximine, a GS inhibitor (Chin et al., 2019). Application of gene amplification technologies could expand the use of human cell lines, such as HEK293, in therapeutic recombinant protein production.

### Future of Recombinant Protein Production in HEK

Host cells have a major impact on the glycosylation profile of the biotherapeutic produced (Goh and Ng, 2017). In the case of NUWIQ®, HEK293-produced rFVIII exhibited improved function and reduced immunogenicity over CHO or BHK-produced rFVIII as it is completely sulfated and devoid of antigenic Neu5Gc or a-Gal epitopes (Sandberg et al., 2012; Kannicht et al., 2013). HEK293 cells exhibit a greater capacity for γ-carboxylation of glutamic acid and sulfation of tyrosine residues as compared to CHO cells. These PTMs are required for therapeutic glycoproteins such as Drotrecogin alfa and recombinant factor IX respectively (Dumont et al., 2015). Although some biotherapeutics are still produced in human cell lines such as HEK293 due to specific PTM requirements, most complex recombinant glycoproteins are produced in CHO.

Amongst the mammalian expression platforms available in the biopharmaceutical industry, Chinese hamster ovary (CHO) cells are used to produce approximately 70% of recombinant biologics, especially monoclonal antibodies (Lalonde and Durocher, 2017). CHO cells are widely used due to their high productivity, ability to be cultured in suspension, in a serum-free, chemically defined cell culture media. In addition, recombinant proteins produced in CHO carry human-like PTMs which improves bioactivity and reduces immunogenicity due to the absence of an α-galactose epitope (Lai et al., 2013).

Compared to HEK, CHO cells are not able to completely reproduce human glycostructures. This is because CHO cells do not express the enzymes β-galactoside α2,6-sialyltransferase, α1,3/4 fucosyl transferase or β-1,4-N-acetylglucosaminyltransferase III, which are expressed in human cell lines (Goh and Ng, 2017). In addition, as they express cytidine monophosphate N-acetylneuraminic acid hydroxylase, glycoproteins produced in CHO carry the potentially immunogenic N-glycolylneuraminic acid (Tejwani et al., 2018). As glycoproteins with desirable glycosylation profiles can exhibit higher potency, stability, half-life and reduced immunogenicity (Tejwani et al., 2018), the advantage of HEK293 as a host cell line lies in the specific glycosylation profile requirement of the biotherapeutic product produced. This limitation in CHO can potentially be overcome by engineering cells capable of producing glycoproteins with desired glycosylation profiles (Tejwani et al., 2018; Nguyen et al., 2021). Required and desirable glycosylation profiles can be engineered by the expression of enzymes found in humans (Nguyen et al., 2021), or the knockout of enzymes absent in humans (Chai et al., 2020). With these developments in CHO cell glycoengineering, we foresee a further decline in the use of HEK293 as host cells for therapeutic recombinant protein production.

## Viral Vector Production in HEK293

HEK293 is commonly used in the production of viral vectors. It is used in the production of adenoviral and adeno-associated viral vectors due to presence of the adenoviral E1A/B genes which provide helper functions during viral vector production. In retroviral vector production, HEK293T is used due to the expression of the SV40 large T-antigen in the cell line. Expression of the T-antigen allows plasmids which carry the SV40 origin of replication to undergo replication when transfected into the cell (DuBridge et al., 1987). It was also reported that expression of the large T-antigen improved lentiviral vector production (Gama-Norton et al., 2011). However, the improved viral titers in HEK293T were not completely attributed to the effect of T-antigen on plasmid replication or transcriptional activity, suggesting that there are other indirect effects and characteristics of the cell line which enables high-titer retroviral vector production (Gama-Norton et al., 2011; Bae et al., 2020).

Early viral vector production was challenging as production was performed by the transient transfection of adherent HEK293 cells in serum-containing media. Recent advancements in transient transfection technologies, cell line and media development have demonstrated that viral vector production can be performed using serum-free suspension-based transfection process in chemically defined cell culture medium. In addition, stable viral vector producer cell lines have been established. Together, these developments have enabled the scalable production of viral vectors in HEK293 cells.

### Current Technologies for Retroviral Vector Production

Retroviral vectors are enveloped, single-stranded RNA viruses. In this family of viruses, γ-retroviruses (γRV) based on the Murine Leukemia Virus and lentiviruses (LV) based on the Human Immunodeficiency Virus type 1, are commonly used in gene therapies (Elsner and Bohne, 2017; Milone and O’Doherty, 2018). These retroviruses are broadly classified according to their genome organisation into “simple” and “complex” retroviruses respectively (Weiss, 1996). Simple retroviruses contain two separate populations of viral RNAs, where viral genomic RNA is distinct from mRNA. In complex retroviruses, viral genomic RNA functions as mRNA (Balvay et al., 2007).

Retroviral vectors are one of the best tools for gene transfer due to its ability to transduce wide range of cell types (Ausubel et al., 2012). Retroviral transduction can stably modify transduced cells as the retroviral RNA genome, which is converted to DNA by the retroviral reverse transcriptase, can be stably integrated into the host cell’s genome. As LV vectors exhibit a less harmful integration profile compared to γRV vectors, they are more widely used in gene therapies (Mitchell et al., 2004; Elsner and Bohne, 2017). Both γRVs and LVs have been used in the production of chimeric antigen receptor (CAR) T-cell therapies (Milone and O’Doherty, 2018). Since 2017, there have been four FDA approved CAR-T therapies, Yescarta, Kymriah, Breyanzi, and Abecma.

For both γRV and LV vector production, different packaging systems have been developed with each successive generation developed aimed at reducing the risk for replication competent viruses (RCV). In γRV production, as the viral genes are non-cytotoxic, packaging cell lines have been developed to facilitate γRV production. In third generation γRV packaging cell lines, *gag-pro-pol* genes, and the envelope protein are stably expressed in separate expression casettes. γRV production is then achieved by transfection of the packaging cell line with a single vector construct carrying the transgene-of-interest, the Ψ packaging signal, and a modified long terminal repeat (LTR) species to further minimize risk of RCV formation (Rodrigues et al., 2011).

Cytotoxicity of LV genes has made it challenging to establish packaging or producer cell lines. Hence, a four-plasmid transient transfection workflow is widely utilised in the production of LV vectors (Ausubel et al., 2012). The plasmids carry: 1) *Gag-Pro-Pol* which encodes viral structural proteins and enzymes; 2) *Rev* which expresses accessory proteins that is essential for viral genome nuclear exportation; 3) *Env* that encodes for the envelope glycoproteins which engage with cell receptors for cellular entry and; 4) Vector genome that carries the gene of interest (Tomás et al., 2018).

Retroviral vectors are typically produced by the transient transfection of adherent HEK293T cells (Loo et al., 2012; Bauler et al., 2020). However, scaling up production from monolayer flasks is time consuming, labour intensive and requires large workspaces for cell cultivation (Rout-Pitt et al., 2018). To improve process scalability, several serum-free suspension-based production methods have been developed (Segura et al., 2007; Ansorge et al., 2009; Bauler et al., 2020). The suspension-based production methods demonstrated comparable volumetric productivities compared to adherent-based production methods (Table 2 and 3). Despite the improvement in process scalability, retroviral vector production remains challenging. There are high costs incurred for good quality transfection-grade DNA and reagents involved (Merten et al., 2016). In addition, the batch-to-batch variability and short production periods has led to the limitation in production of high-titer and consistent viral vectors (McCarron et al., 2016).

**TABLE 2:** Comparison of various γRV production methods.

Reference	Ghani et al. (2007)	Loew et al. (2010)	Loo et al. (2012)	Ghani et al. (2019)
Production type	Transient, Packaging cell line	Packaging cell line for stable production	Transient	Stable
Culture format	Suspension	Adherent	Adherent	Adherent. Parental cell line can be suspension adapted Ghani et al. (2007)
Cell culture media used	H-SFM derived medium with 0.1% lipid mixture, 0.1% bovine serum albumin, 0.1% Pluronic F68	DMEM +10% heat inacitvated Fetal calf serum	DMEM +10% FBS	DMEM +10% FBS
Cells	293SF	HEK293T	HEK293T	293Vec
Transfection method	Calcium phosphate	Lipofection, TransIt293 reagent	Calcium phosphate	NA
Titer	4E7 IU/ml	1E6 IP/mL	1.5E7 IU/ml	2E7 IU/ml for 3 months
Titration method	GFP expression of transduced HT-1080 cells by flow cytometry	GFP expression of transduced HT-1080 cells by flow cytometry	GFP expression of transduced HT-1080 or NIH3T3 cells by flow cytometry	GFP expression of transduced HT-1080 cells by flow cytometry
Production scale tested	Shake flasks	T-Flasks	Wave cell bag with Fibra-Cel disks	10 cm cell culture dishes
Type of vector	SIN γ-retroviral vectors	SIN γ-retroviral vectors	SIN γ-retroviral vectors	SIN γ-retroviral vectors
Envelope protein/Pseudotype	Amphotropic envelope	GALV	GALV and RD114	RD114

**TABLE 3:** Comparison of various LV production methods.

Reference	Bauler et al. (2020)		Rout-Pitt et al. (2018)	Ausubel et al. (2012)	Segura et al. (2007)	Tomás et al. (2018)	Chen at al., (2020)
Production type	Transient	Transient	Transient	Transient	Transient	Stable constititive	Stable inducible
Culture format	Adherent	Suspension	Adherent	Adherent	Suspension	Adherent	Suspension
Cell culture media used	DMEM +10% FBS +2 mM L-alanyl-L glutamine	Freestyle 293	DMEM, 10% FCS, 10 units/ml Penicillin, 10 μg/ml Streptomycin	DMEM supplemented with 10% FBS, 1% sodium pyruvate, and 1% glutamine	Freestyle	DMEM w/10% FBS	Unspecified ‘Serum-Free culture medium'
Cells	HEK293T	HEK293T	HEK293T	HEK293T	HEK293E-6E	HEK293T	HEK293T
Transfection method	PEIpro	PEIpro	Calcium phosphate	Calcium phosphate	25-kDa Linear PEI	N.A	N.A.
Titer	1.5E8 TU/mL	8.2E7 TU/mL	1E8–1E9 TU/mL	5E7-3E8 TU/mL	1E6 IU/ml	1E6 TU/mL/Day, Stable for >2 months	1E7 TU/mL, Stable for ∼2 weeks
Titration method	ddPCR of transduced HOS cells	ddPCR of transduced HOS cells	RT-PCR	GFP expression of transduced HT-1080 cells was measured by flow cytometry	GFP expression of transduced HEK293E-6E cells by flow cytometry	GFP fluorescence by flow cytometry of transduced HEK293T	qPCR of transduced A3.01 cells
Production scale	Cell factory	5L Erlenmeyer shake flask	Cell factory	CellSTACK - 10 Chamber	3L STB	Tissue culture flasks	2L Erlenmeyer flasks
Type of vector	Third-generation lentiviral vector	Third-generation lentiviral vector	Second-generation lentiviral vector	Third-generation lentiviral vector	Third-generation lentiviral vector	Third-generation lentiviral vector	Third-generation lentiviral vector
Envelope protein/Pseudotype	VSV-G	VSV-G	VSV-G	VSV-G	VSV-G	4070A	VSV-G

#### Future of Retroviral Vector Production

Stable producer cell lines have been generated to improve the scalability of γRV and LV production. Stable, suspension-adapted producer cell lines developed from packaging cell lines could produce yRVs in a serum-free medium have been developed to improve process scalability (Ghani et al., 2007). Subsequently, yRVs producer cell lines for the safer, self-inactivating (SIN) yRVs have also been developed (Loew et al., 2010; Ghani et al., 2019). SIN vectors were first developed by the modification of the U3 sequences of the 3 viral LTR, resulting in the loss of viral enhancer and promoter sequences upon integration in the transduced cell (Yu et al., 1986) This reduces the risk of RCV formation as the viral promoter and enhancers are absent, thus preventing vector mobilisation. In addition, when coupled with the use of weak internal promoters to drive the expression of the gene of interest, risk genotoxicity could be reduced (Modlich et al., 2006; Maetzig et al., 2011).

Stable LV producer cell line are challenging to develop due to the cytotoxicity of the genes required. This can be overcome by using less-cytotoxic viral protease mutants or envelope proteins (Tomás et al., 2018) or the use of inducible expression systems (Stewart et al., 2009; Manceur et al., 2017; Chen et al., 2020). The LentiPro26 stable lentiviral vector producer cell lines was developed by Tomás et al., demonstrated stable, constitutive LV production over a period of up to 2 months while maintaining a volumetric titer comparable to existing transient-transfection based production methods (Tomás et al., 2018). Chen et al., demonstrated that stable producer cell lines can be rapidly generated by the transfection of a single DNA construct carrying all required lentiviral vector components, cutting down the time taken to generate and identify stable producing clones form 6 months in the LentiPro26 system (Tomás et al., 2018), to approximately 4 months (Chen et al., 2020). Further developments in stable γRV and LV production systems would improve γRV and LV productivities to meet the growing demand of these vectors in clinical gene therapy applications.

### Current Technologies for Adeno-Associated Virus Production in HEK

Adeno-associated viruses (AAV) are non-enveloped, single-stranded DNA viruses which have shown safety and efficacy as gene therapy vectors (Naso et al., 2017). Since 2012, there have been three approved rAAV gene therapies, namely Glybera, Luxturna, and, most recently, Zolgensma.

Recombinant AAVs (rAAVs) are typically produced via the transient-transfection of adherent HEK293 cells with three plasmids containing: 1) Adenoviral helper factors, E4, E2a, and VARNA; 2) Adeno-associated virus *rep* and *cap* genes; and 3) Cargo gene flanked by AAV ITR sequences (Naso et al., 2017). HEK293 cells are most frequently used for rAAV production due to the expression of adenoviral E1a/b genes (Graham et al., 1977) which are essential for AAV production (Richardson and Westphal, 1984). Although the HeLa cell line was used for rAAV production in early studies (Tratschin et al., 1984), HEK293 based cell lines are typically favored due to the expression of the adenoviral viral helper genes, E1A/B, which improves rAAV titers during production.

#### Helper-Virus Based rAAV Production/HSV Production System in HEK293

rAAVs can also be produced with the use of helper viruses (Clark, 2002). Wild-type AAVs require co-infection with adenoviruses to undergo productive infection. Early rAAV production used a helper-virus production system whereby, rAAV production is achieved by the infection of host cells with recombinant helper viruses carrying the AAV *rep*, *cap*, and cargo genes (Clark, 2002). Typically, two recombinant viruses are used, one carrying the *rep* and *cap* genes, and another carrying the cargo genes. This minimizes recombination, thus reducing the risk of wild-type AAV formation (Clark, 2002). The use of helper-virus based rAAV production systems are not favoured due to the need to separate the helper-virus from the rAAVs during purification, and the need to demonstrate the absence of replication-competent viruses from the purified.

#### Future of rAAV Production Technologies in HEK293

Although the production of rAAVs for the above therapeutics were performed in adherent-based systems, suspension-based rAAV production technologies have been also developed recently. rAAV production have been demonstrated in suspension adapted HEK293 cell lines with yields comparable to existing adherent-based production systems (Table 4) (Lock et al., 2010; Chahal et al., 2014; Grieger et al., 2016; Blessing et al., 2018; Strobel et al., 2019). With a growing number of suspension-based methods used for the production of rAAV therapeutics in clinical trials (Clément and Grieger, 2016), we forsee the shift away from adherent-based production methods in favor of the more scalable, suspension-based production methods.

**TABLE 4:** Comparison of various rAAV production methods.

Reference	Strobel et al. (2019)	Blessing et al. (2018)	Grieger et al. (2016)	Chahal et al. (2014)	Lock et al. (2010)
Culture format	Adherent	Suspension	Suspension	Suspension	Adherent
Cell culture media used	DMEM +5%FBS	F17	F17, CDM4HEK293	SFM4Transfx-293 Medium	DMEM +10% FBS
Cells	HEK293	HEKExpress	HEK293	HEK293SF-3F6	HEK293
Transfection method	Calcium phosphate	25-kDa Linear PEI	PEI Max	25-kDa Linear PEI	PEI Max
Production scale	Cell Disc	TubeSpin 600	20L WAVE Bioreactor	3L STB	CellSTACK - 10 Chamber
Titer	AAV2: 1.2–4 x 10^13^/vg/L	AAV8: 2.1 × 10^11^/L	AAV2: 2.3 × 10^13^/L	AAV2: 2.1 × 10^13^/L	AAV7: 2.5 × 10^13^/L
		AAV9: 3 × 10^11^/L		AAV6: 2 × 10^12^/L	AAV6: 1.2 × 10^12^/L
					AAV8: 1.38 × 10^14^/L
qPCR titering region	CMV	ITR	GFP	CMV	PolyA

Stable producer cell lines have also been developed to improve the scalability of rAAV production. CEVEC pharmaceuticals have developed ELEVECTA® producer cell lines where AAV production genes are stably expressed in CEVEC’s amniocyte production (CAP) cells where gene expression is controlled by an inducible promoter. Scalable rAAV production is achieved simply by induction, without the use of helper viruses, transfection reagents or plasmids (CEVEC, 2020).

While rAAVs are commonly produced in HEK293-based systems, baculovirus-based systems have been developed showing comparable rAAV titers. The use of the baculovirus expression vector system for rAAV production was first described in 2002 (Urabe et al., 2002) where rAAV production was achieved with the co-infection of *Spodoptera frugiperda* (Sf9) cells with three recombinant baculoviruses, a Rep-baculovirus, a Cap-baculovirus, and a Cargo ITR baculovirus, with the helper functions provided for by the baculovirus.

The OneBac rAAV production systems were developed to improve the scalability of the production system by reducing the number of required recombinant baculoviruses for production from three to one. This was achieved by generating Sf9 cell lines stably expressing the AAV *rep* and *cap* genes (Mietzsch et al., 2014, 2015, 2017). This resulted in a more scalable production system with improved titers over HEK293-based systems. Although the systems described demonstrated similar functionality between Sf9-produced and HEK293-produced rAAVs, a recent study identified the differences between rAAVs produced in both systems (Rumachik et al., 2020). The rAAV capsids produced in Sf9 cells had different post translational modifications compared to those produced in HEK293 (Rumachik et al., 2020). Genomes of rAAVs produced in HEK293 cells exhibited methylation patterns indicative of increased vector potency (Rumachik et al., 2020). Although only serotypes AAV1 and AAV8 were tested, this suggests that HEK293-produced rAAVs are preferred due to the improved vector potency. Unless future serotypes assessed demonstrates that Sf9-produced rAAVs could have improved vector potency, rAAV production in HEK293 would be preferred.

### Production of Adenoviral-Vectored Vaccines and Oncolytic Adenoviruses

Adenoviruses (AdV) are non-enveloped, double-stranded DNA viruses. It was first used as a gene therapy vector as they possess high genomic capacity, high transduction efficiency and were non-integrating with high epichromosomal persistence (Bulcha et al., 2021). However, their applicability is limited in gene therapies limited due to a fatal systemic inflammatory response following AdV gene transfer (Raper et al., 2003). Although the use of AdVs in gene therapies have declined due to this unfortunate incident, AdVs have seen a resurgent use in applications where an immunological response is desired. The capacity to induce a strong immune response has led to the development of vaccine candidates for infectious diseases and cancer immunotherapies (Mendonça et al., 2021). AdVs now constitute majority of viral vectors in clinical trials with its predominant application as vaccines or in cancer therapies (Bulcha et al., 2021).

The COVID-19 pandemic drove the rapid development of adenoviral vectored vaccines and their eventual emergency use (Mendonça et al., 2021). Of the three adenoviral vectored vaccines approved for emergency use by the World Health Organisation (as of August 2021), Ad5-nCOV(Zhu et al., 2020) and ChAdOX1-nCoV (Doremalen et al., 2020) are produced in HEK293 cells, while Ad26. COV2-S is produced in PER. C6 cells (Bos et al., 2020). These cells are utilised to produce of AdV vectors due to the need for *trans*-complementation of viral vector genes in the production cell lines.

First generation AdV vectors are produced by the substitution of the E1 and/or E3 regions with an expression cassette. E1 gene products which are necessary for viral production, are provided for in trans in production cell lines such as HEK293 or PER. C6. Products from the E3 gene region are not essential for viral replication and hence, does not need to be complemented. Due to the expression of other viral proteins, first-generation adenoviral vectors provoke an immune response. In the case of a vaccine, the immune response elicited is particularly advantageous (Danthinne and Imperiale, 2000).

Subsequent generation of AdV vectors involve the removal of viral genes to minimize the immune response elicited from the use of these vectors *in vivo*. In second generation AdV vectors, additional genes required for viral replication have been inactivated. Virus production is performed in cell lines expressing the E2 and/or E4 gene products. Finally, in the third class of “gutless adenovirus”, all viral genes have been removed except for the *cis*-acting DNA sequences required for viral DNA replication and packaging (Danthinne and Imperiale, 2000). Advancements to these AdV production systems involve modifications to reduce sequence homology and thus, reducing the risk of generating wild-type AdV through random recombination events (Tripodi et al., 2021) or modifications to minimize transgene expression during viral propagation to improve viral production (Danthinne and Imperiale, 2000; Tripodi et al., 2021).

#### Other Production Systems for Adeno-Associated Viruses and Adenoviruses

Production of AdVs and AAVs require the *trans*-complementation of the adenoviral E1 genes in HEK293 cells. However, as HEK293 cells were generated using sheared AdV genomic DNA, the E1 gene carried DNA sequences with high homology to wild-type AdVs. Production of first-generation E1-deleted adenoviral vectors in HEK293 cells resulted in the generation of RCV with E1 sequences regained, presumably resulting from homologous recombination (Hehir et al., 1996). Despite the use of E1 and E3 deleted adenoviral vectors, formation of RCVs could not be eliminated (Lochmller et al., 1994).

To reduce homology, using a defined E1 gene region to transform a cell line would reduce the risk of generating wild-type AdV through random recombination events during AdV production. The PER. C6 cell line was established by the transfection of human embryonic retinal cells with a defined region of the Adenovirus type 5 E1 gene under the control of the human phosphoglycerate kinase promoter (Fallaux et al., 1998). Production of E1-deleted adenoviral vectors in the PER. C6 cells did not result in the generation of replication competent viruses, allowing for the cost-effect production of AdV vectors. Akin to PER. C6 cells, CEVEC’s CAP cells were immortalized with a vector containing E1 and pIX of AdV 5, for the purpose of high titer production of replication-deficient adenoviral vectors without the accidental formation of RCVs (Schiedner et al., 2000). AdV production might be preferred in these cell lines over HEK293 due to the decreased risk of accidental RCV formation.

## Conclusion

The human cell line, HEK293, have emerged to be the main production platform for therapeutic viral vectors. Advancements in cell line developments would see more HEK293 clones or derivatives developed for growth in a serum-free suspension medium for high-titer viral vector production. Current examples include the commercially available LV and AAV vector production workflow offered by Thermo Fisher Scientific, where their HEK293-derived Viral Production Cells (VPC) are used in the LV-MAX^TM^ system and the upcoming VPC 2.0 used in their prototype AAV-MAX system.

Future of viral vector production technologies leans towards development of stable cell lines for AAV, LV and yRV production. Such processes enable the production of viral vectors without the need to use costly reagents required for transfection. In stable producer cell lines, process developments in CHO resulted in a 10–20 fold improvement in glycoprotein production (Hacker et al., 2009; Kunert and Reinhart, 2016). Advancements in HEK293 cell culture media development and process technologies could lead to a similar improvement of viral vector production in stable production systems.
